# The relationship between dimensions of empathy and symptoms of depression among university students during the COVID-19 pandemic: A network analysis

**DOI:** 10.3389/fpubh.2022.1034119

**Published:** 2022-12-22

**Authors:** Jiayi Li, Chang Liu, Teresa Wulandari, Panhui Wang, Kuiliang Li, Lei Ren, Xufeng Liu

**Affiliations:** ^1^Military Medical Psychology School, Fourth Military Medical University, Xi'an, China; ^2^BrainPark, Turner Institute for Brain and Mental Health and School of Psychological Sciences, Monash University, Clayton, VI, Australia; ^3^Department of Developmental Psychology for Armyman, School of Medical Psychology, Army Medical University, Chongqing, China

**Keywords:** COVID-19, empathy, depression, network analysis, symptom level, bridge nodes, sex differences

## Abstract

**Background:**

The relationship between different dimensions of empathy and individual symptoms of depression during the COVID-19 pandemic remains unclear, despite the established link between empathy and depression. The network analysis offers a novel framework for visualizing the association between empathy and depression as a complex system consisting of interacting nodes. In this study, we investigated the nuanced associations between different dimensions of empathy and individual symptoms of depression using a network model during the pandemic.

**Methods:**

1,177 students completed the Chinese version of the Interpersonal Reactivity Index (IRI), measuring dimensions of empathy, and the Chinese version of the Patient Health Questionnaire-9 (PHQ-9), measuring symptoms of depression. First, we investigated the nuanced associations between different dimensions of empathy and individual depressive symptoms. Then, we calculated the bridge expected influence to examine how different dimensions of empathy may activate or deactivate the symptoms of depression cluster. Finally, we conducted a network comparison test to explore whether network characteristics such as empathy-depression edges and bridge nodes differed between genders.

**Results:**

First, our findings showed that *personal distress* was positively linked to symptoms of depression. These symptoms involved *psychomotor agitation or retardation* (edge weight = 0.18), *sad mood* (edge weight = 0.12)*, trouble with concentrating* (edge weight = 0.11), and *guilt* (edge weight = 0.10). *Perspective-taking* was found to be negatively correlated with *trouble with concentrating* (edge weight = −0.11). *Empathic concern* was negatively associated with *suicidal thoughts* (edge weight = −0.10) and *psychomotor agitation* or *retardation* (edge weight = −0.08). *Fantasy* was not connected with any symptoms of depression. Second, *personal distress* and *empathic concern* were the most positive and negative influential nodes that bridged empathy and depression (values of bridge expected influence were 0.51 and −0.19 and values of predictability were 0.24 and 0.24, respectively). The estimates of the bridge expected influence on the nodes were adequately stable (correlation stability coefficient = 0.75). Finally, no sex differences in the studied network characteristics were observed.

**Conclusions:**

This study applied network analysis to reveal potential pathways between different dimensions of empathy and individual symptoms of depression. The findings supported the existing theoretical system and contribute to the theoretical mechanism. We have also made efforts to suggest interventions and preventions based on *personal distress* and *empathic concern*, the two most important dimensions of empathy for depressive symptoms. These efforts may help Chinese university students to adopt better practical methods to overcome symptoms of depression during the COVID-19 pandemic.

## 1. Introduction

The emergence of the COVID-19 pandemic and related restriction measures have created a social alienation lifestyle and multiple psychological stressors. These transitions have exacerbated mental health concerns among the general population, particularly symptoms of depression. Symptoms of depression are characterized by *anhedonia, sad mood, trouble with sleeping, fatigue, eating problems, guilt, trouble with concentrating, psychomotor agitation or retardation*, and *suicidal thoughts* (1). These symptoms have been shown to affect long-term cognitive performance and quality of life (2). Therefore, exploring possible risks, protective factors, and ways to alleviate symptoms of depression is a public health priority (3).

*Empathy*, the ability of one to share and understand the internal states of others (4), has been identified as a key factor in understanding depression (5, 6). Theoretically, Zahnwaxler and colleagues proposed that empathy may exaggerate a sense of responsibility for what happens to others (e.g., parents' suffering). It then produces an uncomfortable pervasive sense of guilt, exacerbating symptoms of depression (7, 8). Further, Tone and Tully (9) argued that empathic neurobiological processes would form personal distress and guilt, after being moderated by intraindividual and interindividual factors. It could then lead toward internalizing problems in the form of anxiety and depression. However, empirical studies found inconsistent results regarding the influence of empathy on depression, before and during the pandemic. On one hand, the overly active empathetic reaction may increase the likelihood of symptoms of depression due to unrealistic guilt from failure of alleviating suffering of others (10, 11). On the other hand, empathy can help people to understand their problems from various perspectives, hence cope better during the pandemic (12). For example, empathy can better buffer adverse outcomes of pressure and promote the demonstration impact of hope (12).

The internal heterogeneity of empathy could partially explain the inconsistency of the reported associations between empathy and depression. *Empathy* is a multi-faceted construct that includes four dimensions. These dimensions are: *perspective taking* (an inclination to adopt other people's psychological opinions spontaneously), *empathic concern* (a feeling of concerned and compassion for the unfortunate of others), *fantasy* (the tendency to realistically imagine the emotions and behaviors of characters in literary or media works), and *personal distress* (the uneasiness and anxiety in the face of tense interpersonal settings) (13). Different dimensions of empathy are related differently to depression (14). For example, *perspective-taking* reflecting the cognitive component of empathy has been shown to predict depression negatively, while *personal distress* reflecting the affective aspect of empathy has been shown to predict depression positively (15).

Depression heterogeneity is another potential reason for the inconsistency. *Depression* is a heterogeneous syndrome characterized by various cognitive, affective, and somatic symptoms (16). Emerging studies have demonstrated that predisposing variables are related differently to co-occurring symptoms (17–20). Thus, it is plausible to assume that different dimensions of empathy may have unique symptom pathways to co-occurring symptoms of depression. For example, *perspective-taking* involves the frontal brain areas, which might explain the *trouble with concentrating* symptom of depression (21). However, *personal distress* may be particularly relevant to the *guilt* symptom (9). Research examining the relationship between empathy and depression has focused almost exclusively on the construct level (using sum scores) despite empirical evidence suggesting that it is critical to analyze specific symptoms of depression. Focusing on a construct level may conceal important or unique symptom pathways linking empathy and depression. Therefore, moving from sum-scores analysis to dimension and symptom-level analysis may facilitate a more valuable understanding of the relationship between the two.

Network analysis is a statistical method especially suitable for dimension and symptom-level analysis. In the network approach, neuropsychological conditions result from direct interactions between symptoms rather than being underpinned by latent variables (22). Under external factors (e.g., psychological and societal mechanisms), some psychopathological constructs may be activated and transmit the effect to other constructs to generate a feedback loop (22). Recently, more studies have shown that network analysis is a valuable tool to reveal complex associations, such as the underlying mechanisms between symptoms of a given neuropsychological condition and its risk factors (11, 23–26). Utilizing partial correlations in network models allows researchers to explore unique pairwise interactions between variables and adjust for multicollinearity and generate hypotheses on potential causal effects (27, 28).

There are two key advantages of using network analysis in the present study. The first is contributing to a related theoretical framework. Network analysis represents a significant innovation to explore and visualize the interplay between different constructs in a bottom-up data-driven manner and contributes to the generation of hypotheses (27). Thus, network analysis may help expand the current theoretical research on depression and empathy from a latent variable level (total score) to a symptom/dimension level. The second is providing the potential targets for related interventions and preventions. Network analysis offers the bridge expected influence index (e.g., the sum value of all connecting edges in the other communities for a specific node) to identify influential nodes that bridge predisposing variables and symptoms of psychopathology. Nodes with high bridge expected influence may more likely confer risks to other communities (29). Therefore, predisposing variables with high bridge centrality indexes may be potential targets for related preventions and interventions.

During the pandemic, symptoms of depression among university students have increased significantly (3), and empathy is regarded as critical for healthy self-regulation (30, 31). Further, although studies have found that females may be more susceptible to empathy and depression (32, 33), whether sex differences play a role between the two constructs remains unclear (21). For instance, *personal distress* could explain why females are more prone to depression but the relationship between *empathic concern* and depression was not different between sexes (21, 34, 35). Additionally, no sex differences were observed in cognitive components of empathy, such as *perspective-taking* (21, 35–37). To shed light on these gaps, we sought to construct an integrated network combining dimensions of empathy and symptoms of depression. There were three aims in the current research: (1) to investigate the relationship between different dimensions of empathy and symptoms of depression; (2) to identify positive and negative influential nodes that bridge empathy and depression by using bridge centrality indexes; and (3) to explore whether there are sex differences in the above network characteristics.

## 2. Methods

### 2.1. Ethics statement

The data collection procedure followed the Declaration of Helsinki and was approved by the Ethics Committee of the First Affiliated Hospital of the Fourth Military Medical University (Project No. KY20202063-F-2).

### 2.2. Participants

We recruited 1,204 Chinese students using a convenience sampling method between 12 March and 20 September 2021. By intentionally constraining the sampling frame, this sampling method is considered generalized to Chinese university students as subpopulations. To avoid the generalizability of the sample being too narrow and external validity being low, we selected universities of different levels and college students of different majors in Shaanxi and Jilin Province of China. All participants provided their consent before completing a questionnaire through a Chinese online platform (Wenjuanxing). We included two honesty check questions (please select “not at all” for this question and please select “describes me very well” for this question) to detect low-quality data. An incorrect response in at least one of the two questions was considered invalid data (*n* = 27). Each item in the questionnaire required a response, hence, there were no missing items in any participants. The final valid data was 1,177 (505 males, 672 females; mean age = 20.38 years; SD = 2.09). Upon completing the questionnaire, participants received one to five Chinese Yuan, values drawn at random.

### 2.3. Measures

#### 2.3.1. Dimensions of empathy

The *Interpersonal Reactivity Index* (IRI) measures empathy and is composed of four dimensions: *perspective-taking, empathic concern, fantasy, and personal distress* (13). Our study used the Chinese version of the IRI. The scale is comprised of 22 items and scored from 0, *does not describe me very well*, to *4, describes me very well* (38). The current sample's Cronbach 's alpha values were as follows: full scale (0.76), *perspective-taking* subscale (0.76), *empathic concern* subscale (0.64), *fantasy* subscale (0.63) and *personal distress* subscale (0.78).

#### 2.3.2. Symptoms of depression

The Patient Health Questionnaire-9 (PHQ-9) is a brief, valid, self-administered screening tool widely used to measure depression (39). The Chinese version of the PHQ-9 was used in the current study (40). It has nine items providing continuous scores of symptoms of depression. Participants reported to what degree each item applies to them on a 4-point Likert scale ranging from 0, *not at all*, to 3, *nearly every day*. The Cronbach 's alpha of PHQ-9 was 0.87 in this study.

### 2.4. Statistical analysis

Statistical analyzes were conducted through R-statistical software. We modeled the empathy-depression network *via* Gaussian graphical models [GGMs; (41)]. All dimensions of empathy and symptoms of depression were depicted as nodes. An edge between any two nodes demonstrated a partial correlation of the two variables after conditioning on all other network variables. This study used Spearman correlations as input when estimating the network structure, which is recommended for non-normally distributed data (28, 42). Due to the high sample size (*n* = 1,177) and the expectation that there may be many weak bridging edges between dimensions of empathy and symptoms of depression, this study used the unregularized model selection approach (42, 43). Following recent recommendations (42), the network estimation in this study was based on the ggmModSelect technique in the R-package *qgraph* (44). The Fruchterman-Reingold algorithm was used for visualizing the layout (45). The network was visualized *via* the R-package *qgraph* (44).

We calculated the bridge expected influence (the sum of edge weights from a given node in one community to another) to identify influential nodes that bridge dimensions of empathy and symptoms of depression (29). Higher bridge expected influence values implied increased cross-cluster connectivity (29). Two communities were pre-defined before analysis: the dimensions of empathy community (four dimensions from the IRI scale) and the symptoms of depression community (nine items from the PHQ-9 scale). The bridge expected influence was calculated through the R-package *networktools* (29). Further, we calculated the mean node predictability to quantify the mean explained variance of the estimated network *via* R-package *mgm* (46).

The accuracy of edge weights was assessed by plotting the 95% confidence interval (with 2,000 bootstrap samples) for each edge within the presented networks and calculating bootstrapped difference tests for each edge weight. The stability of bridge expected influence was evaluated by computing the correlation stability (CS)-coefficient *via* a case-dropping bootstrap approach (with 2,000 bootstrap samples) and computing bootstrapped difference tests for bridge expected influence. According to a previous recommendation (47), the ideal CS-coefficient is above 0.5 and should be at least 0.25. The procedures mentioned above were conducted *via* the R-package *bootnet* (47).

Finally, we used the R-package *NetworkComparisonTest* to compare whether sex differences existed in the empathy-depression network (permutations = 1,000; 48). Specifically, we compared global strength (summed edge weights of the networks), between-community edge weight and bridge expected influence of the empathy-depression network in male and female participants. Since we had no prior hypotheses about differences in edges, corrections for multiple comparisons were not used when testing them in the current exploratory setting (48).

## 3. Results

### 3.1. Sample characteristic and descriptive statistics of variables

Table 1 displayed the sample characteristics. Table 2 showed the mean scores and standard deviations for each variable selected in the present network.

**Table 1:** Sample characteristics (*n* = 1,177).

**Variables**	***N* (%)/Mean (SD)**
Age	20.38 (2.09)
Female	672 (57.09)
Only child	480 (40.78)
University grade	
First grade	2 (0.02)
Second grade	20 (1.70)
Third grade	27 (2.29)
Fourth grade	1,042 (88.53)
Fifth grade and above	86 (7.31)
IRI-22 total	51.42 (10.00)
PHQ-9 total	6.36 (4.38)

**Table 2:** Abbreviation, mean scores and standard deviations for each variable selected in the present empathy-depression network.

**Variables**	**Abbreviation**	**Mean (SD)**
Dimensions of empathy		
Perspective taking	PT	16.69 (3.84)
Fantasy	F	20.78 (4.11)
Empathic concern	EC	22.31 (3.79)
Personal distress	PD	13.64 (4.25)
Symptoms of depression		
Anhedonia	D1	0.81 (0.66)
Sad mood	D2	0.75 (0.60)
Trouble sleeping	D3	0.82 (0.80)
Fatigue	D4	0.93 (0.72)
Eating problems	D5	0.73 (0.77)
Guilt	D6	0.73 (0.76)
Trouble concentrating	D7	0.90 (0.76)
Psychomotor agitation/retardation	D8	0.44 (0.63)
Suicidal thoughts	D9	0.24 (0.50)

### 3.2. Relations between dimensions of empathy and symptoms of depression

**Figure 1:**
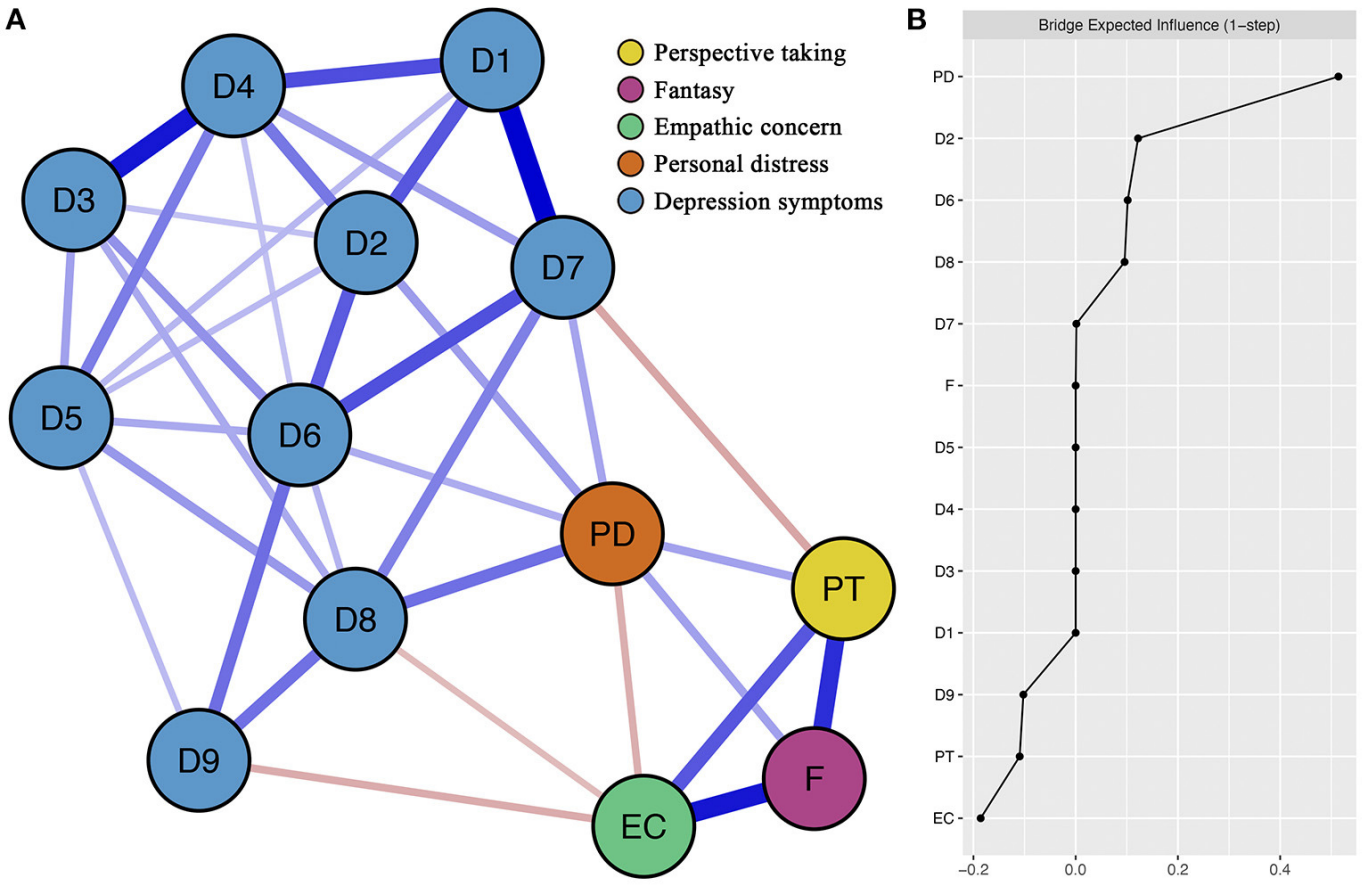
Network structure of different dimensions of empathy and different symptoms of depression. **(A)** Blue edges represent positive correlations, and red edges represent negative correlations. The thickness of the edge reflects the magnitude of the correlation. The text of variables selected in the network can be seen in Table 2. **(B)** Centrality plot depicting the bridge expected influence of each variable in the network.

Figure 1A presented the empathy-depression network. There were 7 of 36 (19.4%) possible between-community edges (four positive edges and three negative edges; weight range from −0.11 to 0.18) within the network. The positive between-community edges were PD (*personal distress*)—D8 (*psychomotor agitation or retardation*; edge weight = 0.18), PD—D2 (*sad mood*; weight = 0.12), PD—D7 (*trouble with concentrating*; weight = 0.11), PD—D6 (*guilt*; weight = 0.10). The negative between-community edges were PT (*perspective-taking*)—D7 (*trouble with concentrating*; weight = −0.11), EC (*empathic concern*)—D9 (*suicidal thoughts*; weight = −0.10), EC—D8 (*psychomotor agitation or retardation*, weight = −0.08). There was no edge between F (*fantasy*) and symptoms of depression. Supplementary Figure 1 showed the bootstrapped 95% confidence interval of edge weights within the empathy-depression network. Supplementary Figure 2 showed the bootstrapped difference test for edge weights.

### 3.3. Bridge expected influence

Figure 1B showed the bridge expected influence values of each node. PD had the highest positive bridge expected influence (value = 0.51). EC and PT had the highest negative bridge expected influences (value = −0.19 and −0.11, respectively). The CS-coefficient of bridge expected influence (value = 0.75) was larger than 0.5, indicating these centrality indexes were adequately stable (Supplementary Figure 3). Supplementary Figure 4 showed the bootstrapped difference tests for node bridge expected influence. Moreover, the results of other centrality indexes (i.e., expected influence, strength, closeness and betweenness) within empathy-depression network can be found in Supplementary Figures 5–7. Mean node predictability of the final empathy-depression network was 0.35. This indicated that on average, 35% of the variance of nodes in the present network can be explained by their neighboring nodes. More details about node predictability can be found in Supplementary Table 1.

### 3.4. Network comparison between genders

We did not find significant sex differences in the network global strength (Global strength [S] = 0.27, male = 5.65, female = 5.38, *p* = 0.31) between-community edges and node bridge expected influence.

## 4. Discussion

There is a lack of understanding of how each dimension of empathy may relate to individual symptoms of depression, especially during the COVID-19 pandemic. In the present study, we addressed this gap by showing specific associations between dimensions of empathy and symptoms of depression *via* network analysis. The findings add to the literature by showing that the related protective and risk factors differed considerably for symptoms of depression (49–55). Further, we calculated the bridge expected influence to detect influential nodes connecting dimensions of empathy and symptoms of depression. Our results indicate that *personal distress* has the highest positive bridge expected influence, while *empathic concern* has the highest negative bridge expected influence. Lastly, we found no sex differences in the structure of the empathy-depression network. These results contribute to the clarity of the empathy-depression theoretical framework and provide potential targets for related preventions and interventions.

### 4.1. Relations between dimensions of empathy and symptoms of depression

In concert with theoretical assumptions by Zahnwaxler et al. (7, 8), this study found that *personal distress* was positively associated with *guilt*. Individuals characterized by *personal distress* may experience excessive self-criticism when viewing aversive events (56), increasing the likelihood of feeling *guilt* during such events (57). The ongoing pandemic has heightened people's psychological distress while also causing them to suffer from experiences of *guilt*. For example, social workers might experience guilt and anxiety when they face people who died from the epidemic (58). Similarly, patients with COVID-19 may feel guilty about being around others and have negative emotional distress due to the stigmatization of the disease (59). Their families and medical staff could also feel guilty for not sharing their pain (60).

Echoing the theoretical model of Tone and Tully (9), our study showed positive relationships between *personal distress* and symptoms of depression, such as *psychomotor agitation or retardation, trouble with concentrating*, and *sad mood*. Individuals prone to *personal distress* and empathic reactions show an inclination to experience physiological hyperarousal and focus their cognitive resources on self-soothing (61). Nevertheless, these individuals are more sensitive to negative emotions in challenging situations (61). As a result, these individuals are more predisposed to the symptoms of depression above. In the context of the pandemic, people with a high level of *personal distress* may pay too much attention to their distress and get caught in negative moods (62).

Compared to *personal distress, perspective-taking* was negatively correlated to *trouble with concentrating*. These findings were consistent with a previous study (63). For instance, attentional shifts that correspond to depression and *perspective-taking* require switching between externally and internally focused viewpoints (63, 64). As such, individuals who are good at *perspective-taking* can switch back to ongoing things that require attention easily during situations that may *cause trouble with concentratin*g. Hence, they experience less *trouble with concentrating* (65).

*Empathic concern* was negatively associated with *psychomotor agitation or retardation* and *suicidal thoughts*. According to the emotion-specificity hypothesis, *psychomotor agitation or retardation* is associated with deficits in emotion recognition (66), which may be due to the low ability of *empathic concern* (67). Meanwhile, the impairment in *empathic concern* reflecting negative ruminations could make people more likely to have thoughts of death (68). As the pandemic has brought an increase in suicides and a decrease in empathy (69), strategies to show more *empathic concern* for others might reduce depression, particularly *suicidal thoughts*, during the continuity of the pandemic (70).

*Fantasy* was not connected with any symptoms of depression. Our result was in line with previous studies, which found no significant association between *fantasy* and depression (71, 72). Indeed, in contrast to the other three dimensions directly related to empathy, *fantasy* does not directly represent empathy (32). Although IRI is considered the most appropriate tool to measure empathy to date, Baron-Cohen and Wheelwright (32) argued that the content measured by the *fantasy* subscale is broader than empathy itself. This discrepancy should be explicitly examined in future research.

### 4.2. Bridge expected influence

In the present network, node bridge expected influence might cast light on the particular role played by different dimensions of empathy in the context of depression. *Personal distress* has the highest positive bridge expected influence suggesting that it is the most positively influential node that bridges empathy and depression. Thus, from a network perspective, *personal distress* is the most critical dimension of empathy in activating risk for symptoms of depression and targeting *personal distress* may be more effective at reducing symptoms of depression than targeting other dimensions of empathy. Mindfulness-based stress reduction (MBSR) is an 8–10 weeks group intervention to develop mindfulness through intensive practice (73). Previous studies have shown that MBSR effectively reduces *personal distress* (74). The significant effects of MBSR on symptoms of depression have been demonstrated in a range of clinical and non-clinical populations (74). Particularly during the epidemic, MBSR has shown promising results for medical staff (75), isolated patients (76), unemployed people (77), and university students (78) to reduce distress and depression. This study supports the scientific application of MBSR and recommends it as an intervention for depression treatment of young adults deeply affected by *personal distress*.

*Empathic concern* has the strongest negative bridge expected influence, indicating that it is the most negatively influential node that bridges empathy and depression. Thus, *empathetic concern* may be the most critical dimension of empathy in activating protection for symptoms of depression. Compassion Cultivation Training (CCT) is an 8-week intervention to enhance compassion and empathy (79). Compared to MBSR, which takes an implicit approach to teaching empathy as a mindfulness practice attitude, CCT takes a more straightforward approach to training empathy. Although both CCT and MBSR can improve the ability of empathy and reduce depression, CCT is especially effective in promoting *empathic concern* and identification with humanity (79). Our results recommend CCT as an effective program to improve *empathic concern* and prevent depression. Incorporating CCT into the psychoeducation program may have a protective effect on the mental health of young adults facing the risk of depression.

### 4.3. Network comparison between genders

Although previous studies have shown that empathy and depression may be affected by sex, we found that sex had no significant influence on the relationship, nor bridge centrality. These results were in concert with previous work (21, 80), which means there is insufficient research to conclude sex differences in the relationship (21). Despite sex having been found to influence empathy and depression, this effect might not extend to the link between the two. Overall, the network structure results' theoretical contribution and the bridge influence's expected clinical implications in this study are suitable for both females and males, and further evidence is needed to draw more convincing conclusions in the future.

### 4.4. Limitations

One key limitation of the current study is the usage of cross-sectional data. We can only estimate undirected networks and cannot determine the direction of edges. Future work could use longitudinal data to ascertain the causality dynamically. Second, all study variables were measured at a group level. Thus, the research conclusions could not be applied to a single individual. Third, most of the participants in this study were graduating university students. Therefore, the conclusions of this study are more applicable to the young adult group facing the pressure of further education and employment under the pandemic, and the generalizability to populations in other age groups or geographical locations should be further verified. Fourth, non-probability sampling strategies were used in this study; probability sampling strategies are recommended in the future to yield an unbiased sample representative of the target population. Fifth, like other existing studies that examine how external field factors may act directly upon symptom networks (e.g., emotion regulation and intolerance of uncertainty) (17, 23, 26, 52, 53), we focused on one particular construct of the external field (i.e., empathy). Future research could add potential covariates (e.g., age, exercise, smoking, and chronic diseases) (50) into the model to further improve its validity. Finally, since there was no pre-pandemic data, the specific impact of the pandemic on the relationship between empathy and depression cannot be fully determined.

## 5. Conclusion

The present study advances the understanding of the relationship between empathy and depression through the network approach. Our results showed that different dimensions of empathy have specific relations with different symptoms of depression. From the network perspective, this study supports previous theoretical models of the relationship between empathy and depression while also discovers more diverse pathways, such as *psychomotor agitation or retardation, trouble with concentrating*, and *sad mood*. These explored pathways provide a new insight for the improvement of theoretical mechanisms related to empathy and depression. Moreover, our findings suggest that preventions and interventions that target *personal distress and empathic concern* of empathy may be promising in reducing depression from the context of empathy during the COVID-19 pandemic.

## Data availability statement

The raw data supporting the conclusions of this article will be made available by the authors, without undue reservation.

## Ethics statement

The studies involving human participants were reviewed and approved by Ethics Committee of the First Affiliated Hospital of the Fourth Military Medical University. The questionnaire was completed online through a Chinese platform (Wenjuanxing) after participants provided written informed consent.

## Author contributions

JL and LR developed study idea and design. JL and PW collected data. JL, CL, LR, and XL wrote the original draft of this manuscript. JL, CL, LR, TW, and KL revised the manuscript. XL, CL, and LR give guidance to the manuscript. All authors contributed to revising subsequent versions of the paper.
