# Supplementary material for: The relationship between dimensions of empathy and symptoms of depression among university students during the COVID-19 pandemic: A network analysis
Source: Front Public Health. 2022 Dec 22;10:1034119. doi: 10.3389/fpubh.2022.1034119 (PMC9813512; doi:10.3389/fpubh.2022.1034119)
Supplement: Supplementary file 1 [file Data_Sheet_1.docx]

**Supplementary Materials**

**The results of empathy-depression network**

1. Figure S1. Accuracy of edge weights
2. Figure S2. Bootstrapped difference test for edge weights
3. Figure S3. Stability of node bridge expected influences
4. Figure S4. Bootstrapped difference test for node bridge expected influences
5. Figure S5. The results of other centrality indexes (i.e., expected influence, strength, closeness and betweenness) within empathy-depression network
6. Figure S6. Stability of node strengths (CS-coefficient = 0.672).
7. Figure S7. Bootstrapped difference test for node strengths
8. Table S1. The node predictability of each variable

Figure S1. Accuracy of edge weights

Note: The red line depicts the sample edge weights and the gray bar depicts the bootstrapped confidence interval.

Figure S2. Bootstrapped difference test for edge weights

*Note*: Gray boxes indicate edge weights that do not differ significantly from one another, while black boxes indicate edge weights that do differ significantly. Blue and red boxes on the diagonal correspond to edge weights with positive and negative correlations, respectively.

Figure S3. Stability of node bridge expected influences

*Note*: The red bar represents the average correlation between node bridge expected influences in the full sample and subsample with the red area depicting the 2.5th quantile to the 97.5th quantile.

Figure S4. Bootstrapped difference test for node bridge expected influences

*Note*: Gray boxes indicate node bridge expected influences that do not differ significantly from one another, while black boxes indicate node bridge expected influences that do differ significantly.


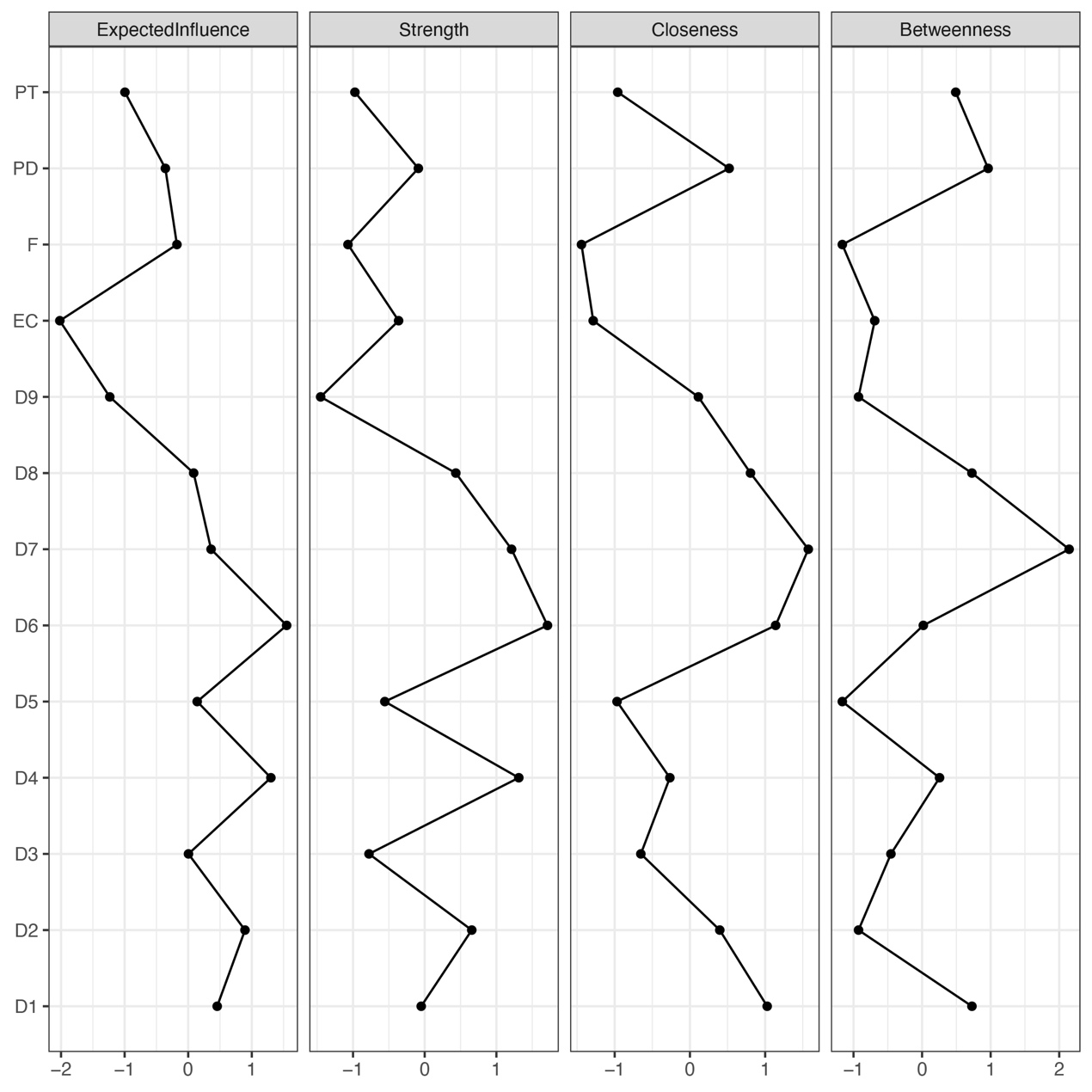


Figure S5. Centrality plot depicting the expected influence, strength, closeness and betweenness of each variable in the network.

Figure S6. Stability of node strengths (CS-coefficient = 0.672).

*Note:* The red bar represents the average correlation between node strengths in the full sample and subsample with the red area depicting the 2.5th quantile to the 97.5th quantile.

Figure S7. Bootstrapped difference test for node strengths

*Note*: Gray boxes indicate node strengths that do not differ significantly from one another, while black boxes indicate node strengths that do differ significantly.

Table S1. The node predictability of each variable

| Variables | Abbreviation | Predictability |
| --- | --- | --- |
| Dimensions of empathy |  |  |
| Perspective taking | PT | 0.21 |
| Fantasy | F | 0.23 |
| Empathic concern | EC | 0.24 |
| Personal distress | PD | 0.24 |
| Symptoms of depression |  |  |
| Anhedonia | D1 | 0.46 |
| Sad mood | D2 | 0.47 |
| Trouble sleeping | D3 | 0.38 |
| Fatigue | D4 | 0.51 |
| Eating problems | D5 | 0.33 |
| Guilt | D6 | 0.49 |
| Trouble concentrating | D7 | 0.45 |
| Psychomotor agitation/retardation | D8 | 0.35 |
| Suicidal thoughts | D9 | 0.27 |
